# Study of Zoonotic Enteric Pathogens of *Atelerix algirus* in Tenerife, Canary Islands, Spain

**DOI:** 10.3389/fvets.2020.579602

**Published:** 2020-10-08

**Authors:** Elena Izquierdo-Rodriguez, Natalia Martin-Carrillo, Basilio Valladares, Pilar Foronda

**Affiliations:** ^1^Instituto Universitario de Enfermedades Tropicales y Salud Pública de Canarias, Universidad de La Laguna, San Cristóbal de La Laguna, Spain; ^2^Departament Obstetricia y Ginecología, Pediatría, Medicina Preventiva y Salud Pública, Toxicología, Medicina Legal y Forense y Parasitología, Universidad de La Laguna, San Cristóbal de La Laguna, Spain

**Keywords:** *Atelerix algirus*, FilmArray gastrointestinal panel, enteropathogens, hedgehog, Canary Islands

## Abstract

*Atelerix algirus* is an invasive species in the Canary Islands (Spain). There are few studies about the zoonotic pathogens this species could be hosting; therefore, this study was focused on analyzing causative agents of diarrhea in humans in feces from hedgehogs. A total of 45 fecal samples obtained in Tenerife (Canary Islands) were analyzed in this study using Biofire FilmArray gastrointestinal panel with an integrated Biofire FilmArray system. Forty-two (93.33%) of the samples presented at least one of the pathogens detected by the panel. The prevalence of four bacteria stands out as for enteropathogenic *Escherichia coli* (71.11%), *Salmonella* (66.67%), *Clostridioides difficile* (33.33%), and *Campylobacter* sp. (22.22%), all of which were widely distributed along Tenerife. Besides, other pathogens were found, *Cryptosporidium* sp. and enterotoxigenic *E. coli* lt/st in 6.66% of the animals, *Shigella*/enteroinvasive *E. coli* in 4.44%, and *Norovirus* GI/GII, *Plesiomonas shigelloides*, and *Vibrio* sp. in 2.22%. Of the hedgehogs, 26.66% were hosting just one pathogen, and the others showed coinfection: 24.44% hosted two, 31.11% hosted three, and 11.11% hosted four or more. The close contact with hedgehogs may imply the transmission of not only one causative agent of diarrhea but also multiple agents, since coinfection is highly prevalent. The lack of management measurements for this animal in the Canary Islands, the common habit of adopting hedgehogs from wildlife without veterinary control, and the fact that most of the hedgehogs studied belonged to highly populated areas imply a high risk of transmission of pathogens to humans.

## Introduction

The Algerian hedgehog, *Atelerix algirus* (Lereboullet, 1842), is an invasive species in the Canary Islands (Spain), located in NW Africa (13°23′-18°8′W and 27°37′-29°24′). Although it is not clear if the arrival of this mammal to the archipelago was accidental or intended, nowadays it seems to have colonized the islands of Fuerteventura, Lanzarote, Gran Canaria, Tenerife, and, most recently, La Palma (1, 2). Not many studies have been done in order to establish *A. algirus* diet or biodiversity impact on the Canary Islands, and only few studies had been carried out studying the zoonotic pathogens that this hedgehog could be hosting around the world, including *Leishmania major* and some species from the *Rickettsiae* family (3, 4). However, a recent study that also took place in Spain found *A. algirus* specimens hosting *Angiostrongylus cantonensis* (5), a parasite highly distributed in rats and mollusk of Northern Tenerife (6, 7).

Due to its appearance and non-aggressive behavior, wild hedgehogs are often kept as pets in the Canarian archipelago, especially by families with children (authors' personal communication). Because of the lack of knowledge about the possible pathogens that *A. algirus* of the Canary Islands could be hosting, the aim of this study was to examine feces of *A. algirus* from Tenerife island, in order to analyze the presence of causative agents of infectious diarrhea in humans.

## Materials and Methods

**Figure 1:**
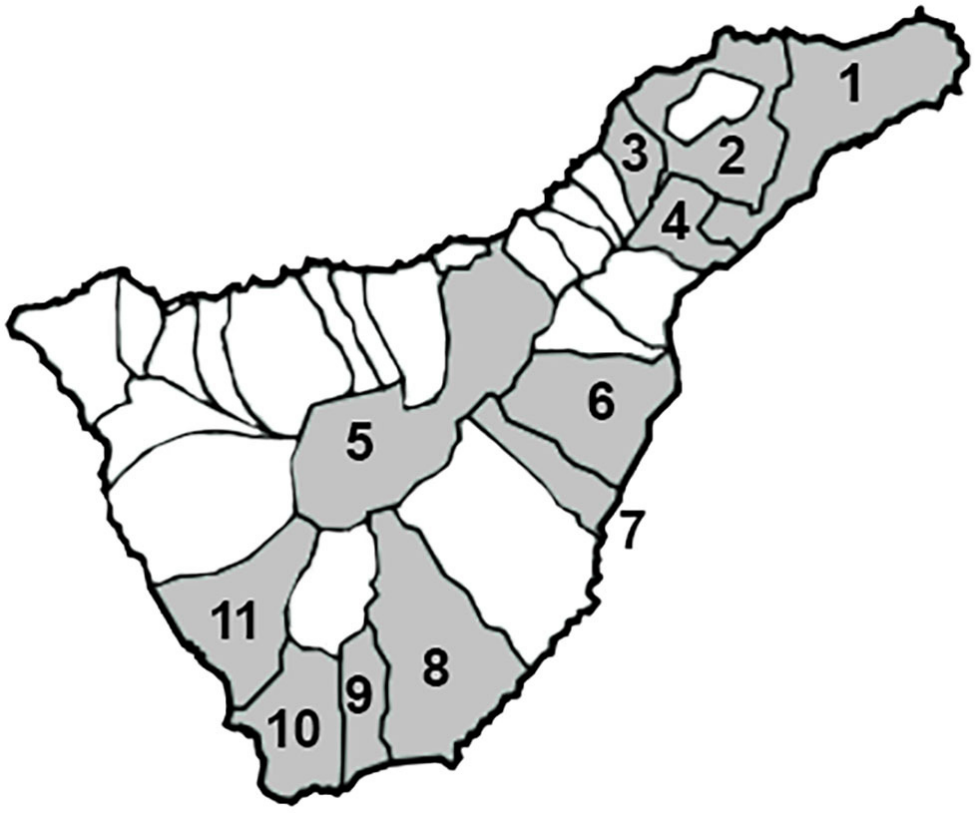
Map showing the municipalities of Tenerife in which hedgehogs were captured (1. Santa Cruz, 2. La Laguna, 3. Tacoronte, 4. El Rosario, 5. La Orotava, 6. Güímar, 7. Fasnia, 8. Granadilla, 9. San Miguel, 10. Arona, 11. Adeje).

A total of 45 feces samples from hedgehogs collected widely along the island of Tenerife in 11 of its 31 municipalities were analyzed in this study (Figure 1). Samples belonged to two different categories, fresh feces from living specimens (*n* = 43) and feces from the intestine of dead animals (*n* = 2). All samples as well as data regarding their provenance were provided by La Tahonilla, a wildlife recovery center of Excmo Cabildo Insular Tenerife. For each fecal sample, no longer than 24 h elapsed from the death of the animal or the collection of the feces and its analysis, and samples were kept refrigerated until the analysis was performed. In the case of dead hedgehogs, after their dissection, feces were collected from the colon and directly analyzed.

All assays on the feces samples were performed at Instituto Universitario de Enfermedades Tropicales y Salud Pública de Canarias (Universidad de La Laguna), using the Biofire FilmArray gastrointestinal panel for the detection of 22 pathogens, which are causative agents of infectious diarrhea in humans, including bacteria, *Campylobacter* (*jejuni, coli, upsaliensis*), *Clostridioides difficile* toxin A/B, *Plesiomonas shigelloides, Salmonella, Yersinia enterocolitica, Vibrio* (*parahaemolyticus, vulnificus, cholerae*), *Escherichia coli* O157, enteroaggregative *E.coli* (EAEC), enteropathogenic *E. coli* (EPEC), enterotoxigenic *E. coli* (ETEC) *lt/st*, Shiga-like toxin-producing *E. coli* (STEC) *stx1/stx2, E. coli* O157, and *Shigella/*Enteroinvasive *E. coli* (EIEC); parasites, *Cryptosporidium, Cyclospora cayetanensis, Entamoeba histolytica*, and *Giardia lamblia*; and viruses including Adenovirus F 40/41, Astrovirus, Norovirus GI/GII, Rotavirus A, and Sapovirus. For each sample, 200 μl was added into each panel, following the manufacturer's instructions, and then analyzed in a Biofire Filmarray integrated system (Biomerieux, France). Results are presented as prevalence and 95% confidence intervals are included.

## Results

The results of this study show a wide distribution of causative agents of diarrhea in humans in hedgehogs of Tenerife, as pathogens were found in every municipality tested, and nearly all of the animals tested, 42 of 45 (93.33%) were positive for at least one pathogen (Table 1 and Figure 1). A total of 11 of the 22 pathogens included in the FilmArray gastrointestinal panel were recorded in the island of Tenerife including nine bacteria, EPEC, enterotoxigenic *E. coli lt/st, Shigella/*Enteroinvasive *E. coli, Salmonella, Campylobacter, Vibrio* sp. (*parahaemolyticys* or *vulnificus*), *P. shigelloides, Clostridioides difficile*, and one protozoa, *Cryptosporidium* sp.; and one virus, Norovirus GI/GII.

**Table 1:** Municipalities where *Atelerix algirus* were captured in Tenerife (Canary Islands, Spain), number of animals per area, and pathogens found.

**Municipality**	**Number of samples analyzed**	**FilmArray Results (number of positive samples)**
La Laguna	11	*Cryptosporidium* sp. (2), enteropathogenic *E. coli* (5), *Campylobacter* sp. (3)*, Salmonella* (7), *Clostridioides difficile* (5), *Shigella/*enteroinvasive *E. coli* (1)
Santa Cruz	8	Enteropathogenic *E. coli* (8), enterotoxigenic *E. coli lt/st* (1)*, Salmonella* (4)*, Clostridioides difficile* (2), *Campylobacter* sp. (1)
Arona	7	*Clostridioides difficile* (2), enteropathogenic *E. coli* (4), enterotoxigenic *E. coli lt/st* (1), *Salmonella* sp. (5), *Campylobacter* sp. (2)
El Rosario	7	*Clostridioides difficile* (1), *Salmonella* sp. (6), enteropathogenic *E. coli* (5), *Campylobacter* sp. (2), enterotoxigenic *E. coli lt/st* (1), *Vibrio* sp. (1)
La Orotava	2	*Cryptosporidium* (1), *Clostridioides difficile* (2)*, Salmonella* sp. (1), enteropathogenic *E. coli* (1), *Plesiomonas shigelloides* (1)
San Miguel	3	*Clostridioides difficile* (1), *Salmonella* sp. (3), enteropathogenic *E. coli* (3)
Granadilla	2	Enteropathogenic *E. coli* (1)*, Campylobacter* sp. (2)
Adeje	2	*Clostridioides difficile* (1)*, Salmonella* sp. (2), enteropathogenic *E. coli* (2)
Tacoronte	1	*Clostridioides difficile* (1)*, Salmonella* sp. (1), enteropathogenic *E. coli*. (1)
Güímar	1	*Salmonella* sp. (1)., enteropathogenic *E. coli* (1), norovirus GI/GII (1)
Fasnia	1	Enteropathogenic *E. coli* (1)
Total	45	*Campylobacter* sp. (10), *Clostridioides difficile* (15), *Plesiomonas shigelloides* (1), *Salmonella* sp. (30), *Vibrio* sp. (1), enteropathogenic *E. coli* (32), enterotoxigenic *E. coli lt/st* (3), *Shigella/*enteroinvasive *E. coli* (1), *Cryptosporidium* (3), Norovirus GI/GII (1)

The high prevalence of four bacteria is remarkable: for EPEC, 32 of the 45 samples were positive (71.11%; CI: 55.68–83.63); for *Salmonella*, 30 of 45 were positive (66.67%; CI: 51.05–79.99); for *Clostridioides difficile* (previously known as *Clostridium difficile*), 15 of 45 were positive (33.33%; 20.00–48.95); and in the case of *Campylobacter* sp., 10 of the 45 samples were positive (22.22%; CI: 11.20–37.09). Among the other pathogens, the prevalence of *Cryptosporidium* sp. and enterotoxigenic *E. coli* lt/st was 6.67% (3 of 45; CI: 13.96–18.27); for *Shigella*/enteroinvasive *E. coli*, Norovirus GI/GII, *P. shigelloides*, and *Vibrio* sp., only 1 of the 45 samples (2.22%; CI: 0.56–11.77) were positive. Most pathogens were highly distributed along the island, although *Cryptosporidium* sp., *Shigella*/enteroinvasive *E. coli, Vibrio* sp., *and P. shigelloides* were only found in Northern municipalities, while Norovirus GI/GII was only detected on the South.

Regarding the infection rate, 12 hedgehogs were hosting just one pathogen (26.66%), 11 hosted two pathogens (24.44%), 14 hosted three (31.11%), and 5 hosted four or more pathogens (11.11%). The most common combination was EPEC and *Salmonella* (51.11%), being 35.55% with other pathogens. It is remarkable that *C. difficile* was always detected in coinfection; however, in no occasion was it detected only with *Salmonella*, as these two bacteria were always found in coinfection with others (Supplementary File).

## Discussion

The introduction of invasive species in a delicate ecosystem like the Canary Islands not only implies a potential danger to the environment, as more than 500 endemic species inhabit the archipelago (8), but also can contribute to the dispersion of different diseases. The results of this study show a wide distribution of causative agents of diarrhea in Tenerife, as the majority of the hedgehogs included in this study were found in the most populated areas of Tenerife, since La Laguna and Santa Cruz are the biggest cities of the island and Arona, San Miguel, and Adeje are highly touristic municipalities. The finding of causative agents of human diarrhea in the hedgehogs from the wildlife of these areas could imply a risk of transmission to humans, specially taking into consideration that in the Canarian archipelago, *A. algirus* is commonly adopted from the environment without any veterinary control.

Regarding the pathogens found in this study, the finding of *Salmonella* sp. in exotic animals of Spain is not unusual, as in 2013 it was found in free-living turtles of Eastern Spain, with a prevalence of 11% (9), although this is the first time this bacterial genus is found in *A. algirus*. The high prevalence of *Salmonella* and *Campylobacter* species in *A. algirus* may be due to the fact that this hedgehog species is mainly an insectivore, although its diet may vary according to availability and weather conditions (10), and some studies have shown that insects can act as carriers of *Salmonella* and *Campylobacter* species (11). The high prevalence of both bacteria found in this study compared to other wildlife species may imply that insects of the Canary Islands are probably carriers of these pathogens and *A. algirus* feeding habits facilitates their infection. Apart from the acute gastroenteritis that both bacteria cause, campylobacteriosis can sometimes lead to long-term sequelae such as Guillain–Barré syndrome, irritable bowel syndrome, or reactive arthritis (9).

The prevalence found in this study regarding *Cryptosporidium* sp. is similar to those found in pigeons of the Canary Islands in 2009, in which 28 wild pigeons were captured in Santa Cruz and its feces were posteriorly analyzed by PCR for the presence of *Cryptosporidium*, with 5.9% of them being positive (12). Further investigations in the presence of *Cryptosporidium* in wildlife species of the Canary Islands should be done in order to prevent risk focuses of cryptosporidiosis, a disease that can cause severe enteritis and malabsorption (13).

The presence of *P. shigelloides* in a hedgehog from La Orotava is also remarkable, a bacterium capable of causing sepsis and meningitis to immunosuppressed hosts, specially babies, in which 12 cases has been described, 6 of whom died due to the infection (14). Besides, the presence of *C. difficile* may be one of the most problematic among the bacteria found in this study, as its infection is transmitted by spores resistant to acid, antibiotics, or even heat and are commonly found in the environment. Also, elderly people, cancer patients and other diseases seem to be risk factors for the susceptibility to this bacterium, which may cause colitis. It is important to note that 40% of the community acquired infections of *C. difficile* requires hospitalization (15). The finding of this bacterium in the feces of animals that can potentially be adopted constitutes a risk of transmission to its owners, which is specially concerning in the case of immunosuppressed individuals.

The finding of *Vibrio* sp. (*parahaemolyticus* or *vulnificus*) in a hedgehog of Tenerife is also interesting, as these bacteria species are usually transmitted by seafood, with *V. vulnificus* being of special concern as it is responsible for more than 95% of seafood-related death in the United States, usually in individuals with liver disease or those who are immunocompromised (16). Besides, both *V. parahaemolyticus* and *V. vulnificus* are the most common causative agents isolated when *Vibrio* spp. are isolated from skin and soft tissue infections, which range from bullous skin lessons to severe necrotizing of the tissue with secondary septicemia (17). In the Canary Islands, one case of skin and soft tissue infection due to *Vibrio* sp. was reported in a patient with cancer and diabetes mellitus type II (17); the presence of this genera of bacteria in the wildlife, especially in a species that usually lives in close contact with humans, could constitute a high risk for immunocompromised individuals, as well as people presenting chronic diseases such as cancer and diabetes.

Regarding the *E. coli* strains found in this study, the prevalence of EPEC found in hedgehogs was considerably higher than other wildlife species studied along mainland Spain (18). That is why hedgehogs should be considered as an important wildlife reservoir of this bacterium, as nearly three quarters of the animals studied hosted EPEC.

In the particular case of Norovirus, one third of the people infected are asymptomatic; however, elderly, children, and immunocompromised people are at greater risk for severe symptoms and complications, such as acute renal failure leading to hemodialysis, cardiac complications including arrhythmias, acute graft organ rejection in transplant recipients, and death (19). The finding of Norovirus GI/GII in animals that can potentially be adopted by families may constitute a risk focus for this viral infection.

Another factor to take into consideration is that hedgehogs present a behavior named anting or anointing, which consist in moistening their spines with saliva (20). The fact that hedgehogs showed high prevalence of gastrointestinal pathogens implies a risk for humans to get in contact with them only by touching the animals, even more taking into consideration that coinfection was commonly found in these animals, so multiple pathogens can be transmitted. Also, further investigations should be done in the islands of the Canary archipelago where *A. algirus* presence has been spotted, as this is the first study on the zoonotic pathogens that this species hosts in the archipelago.

In mainland Spain, two species of hedgehogs inhabiting the country, *Atelerix albiventris* (Wagner, 1841) and *Hemiechinus auratus* (Gmelin, 1770), are included in the catalog of exotic invasive species (RD 63/2013, de 2 de Agosto) (21), which allows the competent authorities to control the population. Management strategies as well as educative measurements should be applied in order to reduce the impact that this species may be having in the Canary archipelago.

In conclusion, the lack of management measurements, the non-controlled selling, and the fact that some of the hedgehogs included in this study belonged to highly populated areas of Tenerife imply a high risk of transmission of pathogens to humans.

## Permission to Reuse and Copyright

The original image from the figure in this article was acquired from https://commons.wikimedia.org/wiki/File:Santa_Cruz_de_Tenerife_-_Mapa_municipal.svg in which the permission to copy, distribute, or modify it is established. It was later edited in photoshop CS6 by Izquierdo-Rodríguez.

## Data Availability Statement

All datasets generated for this study are included in the article/Supplementary Material.

## Ethics Statement

Ethical review and approval was not required for the animal study because we analyzed fresh feces from living specimens and from dead animals donated by an Animal Recovery Center.

## Author Contributions

EI-R and NM-C were responsible for the collection of the samples as well as the analysis. PF coordinated the relationships between the wildlife recovery center and the laboratory, as well as supervised the analysis and the writing of the article. BV and PF obtained the funding for the study and supervised the work. EI-R did the main writing of the article. All authors have read and approved the final manuscript.

## Conflict of Interest

The authors declare that the research was conducted in the absence of any commercial or financial relationships that could be construed as a potential conflict of interest.
